# Application of Ultrasonic Array Method for the Inspection of TC18 Addictive Manufacturing Titanium Alloy

**DOI:** 10.3390/s19204371

**Published:** 2019-10-10

**Authors:** Wentao Li, Zhenggan Zhou, Yang Li

**Affiliations:** 1 School of Mechanical Engineering and Automation, Beihang University, Beijing 100083, China; 2 The Collaborative Innovation Center for Advanced Aero-Engine (CICAAE), Beijing 100083, China

**Keywords:** ultrasonic array, additive manufacturing (AM), TC18 titanium alloy, total focusing method, annular array

## Abstract

Ultrasonic arrays have been investigated for inspecting the quality of special materials. Unfortunately, non-destructive testing and evaluation (NDT&E) of internal defects in additive manufacturing (AM) materials are difficult due to the anisotropy and the coarse grain. To solve the problem, this paper brings forward research on the inspection of TC18 AM titanium alloy products using an ultrasonic array. Firstly, a three-dimensional acoustic field distribution of different ultrasonic array transducers is established to design an optimal detection solution for an AM titanium alloy. Then, a total focusing method (TFM) for the ultrasonic annular array transducer is proposed and its imaging method is analyzed. Besides, the relation between ultrasonic group velocities in a TC18 AM specimen with different propagating angles is measured using the full matrix capture (FMC) method. Based on the measurements, the anisotropy of the AM titanium alloy is discussed and the TFM algorithm of annular array is optimized as well. Finally, C-scan experiments are conducted on the specimen with a height of 55 mm using the linear ultrasonic array transducer of the conventional focusing method and the TFM of annular array transducer, respectively. The results show that the TFM of annular array has higher accuracy in quantifying the defects of flat bottom holes and transverse holes with a diameter of 0.8 mm. In addition, the detection results of different forming directions are analyzed and the 3D imaging of defects in the specimen is realized based on FMC data. The TFM of annular array is an innovative ultrasonic testing technology with high resolution for AM titanium alloy products.

## 1. Introduction

Additive manufacturing (AM), an advanced manufacturing technique with excellent designability and high material utilization, is widely used for rapid prototyping of the complex titanium alloy components in aerospace [1,2]. The AM technology of titanium alloy is involved in the complex physical process of material melting and forming [3]. Hence, many types of defects such as pores, holes, and cracks, easily occurring in the manufacturing process, have influence on the strength of the AM material and limit the application of AM technology in key components [4,5]. However, the AM titanium alloy has obvious differences in velocity and attenuation compared with the conventional titanium alloy, which may lead to problems such as poor signal to noise ratio (SNR), low accuracy, shallow detection depth, and even undetectability [6,7,8]. Therefore, it is very important to find out an effective NDT method for the AM material to ensure the reliability of important equipment in the high-end manufacturing field and to develop AM technologies.

At present, the ultrasonic NDT method is usually used to test the quality of titanium alloy parts [9]. Unfortunately, AM titanium alloy products are inhomogeneous and grain coarsening due to the special manufacturing process, which could lead to wave distortion and strong scattering, makes it hard to detect the internal defects using conventional monolithic transducers. Zeltmann et al., using a conventional ultrasonic immersion probe with a frequency of 5 MHz and a focal length of 25.4 mm, failed to detect the defects embedded in the additive specimen, but these defects could affect the specimen performance under fatigue loading conditions [7]. Cerniglia et al. utilized the laser-generated ultrasonic wave to detect the surface flaws in a single layer, which was confirmed by an ultra-high sensitivity X-ray technique. However, the system can only detect near-surface defects, and the cost of the equipment is quite high [10]. The national aeronautics and space administration (NASA) research center introduced the ultrasonic phase array used for testing embedded voids or weak deposition layers of 2219 aluminum electron-beam freeform fabrication parts [11]. An ultrasonic array is a multi-channel ultrasonic technology that has the characteristics of higher detection accuracy and faster detection speed, usually used in NDT of complex structures and special materials in different fields such as aerospace, nuclear power, petroleum, and other field equipment [12,13,14]. However, there are few researchers focusing on the detection of AM titanium alloy material’s quality using ultrasonic array technique. On the one hand, the material properties of the AM titanium alloy will lead to wave beam dispersion and distortion. On the other hand, the attenuation of different positions on the same AM titanium alloy surface is quite different, making it difficult to detect with the backwall echo method, so strong acoustic beam energy is required when using the defect echo method. Therefore, it is of great significance to research reliable and accurate ultrasonic array testing technology to evaluate the performance of AM titanium alloy structures.

The accurate detection of anisotropic heterogeneous materials and other high attenuation materials is often a problem with conventional linear phased arrays because of the complexity of body-wave propagation and the asymmetry of the acoustic field. In this paper, a three-dimensional acoustic field model of three common ultrasonic array transducers is established. It is found that the annular array transducer can produce better penetration depth and spatial resolution with fewer array elements, and the acoustic field is fully symmetrical in space and it is more suitable for the automatic scanning detection. In addition, a new TFM algorithm for annular array transducer is proposed in this paper. In this method, the data reconstruction area infinitely focuses along the transducer axis, which makes the beam energy more powerful at different depths. At the same time, the acoustic characteristics of TC18 AM titanium alloy are analyzed using different ultrasonic testing methods and the TFM algorithm is optimized by the measured results of the group velocity. Then, a real-time TFM C-scan detection system for annular array transducers is developed based on the CUDA parallel method. Finally, experiments based on the dynamic depth focusing (DDF) algorithm and the TFM algorithm are conducted. The results show that the annular array TFM algorithm for AM titanium alloy has better detection results compared with the conventional focusing method. 

## 2. Theory and Numerical Model

### 2.1. Acoustic Field Distribution of Ultrasonic Array

The synthetic beam energy of ultrasonic array is an important factor affecting the testing result because of the high scattering and attenuation material properties of AM titanium alloy. Therefore, the three-dimensional acoustic field model is established to study the synthetic beam energy distribution of commonly used array transducers. Ultrasonic waves are a kind of mechanical wave, which are the propagation of pressure, displacement, and energy, and can be analyzed by elastic wave dynamics. The elastic wave displacement field in an isotropic medium can be expressed as [15]:

(1) $$\nabla^{2}P(x,t)-\frac{1}{c^{2}}\frac{\partial^{2}P(x,t)}{\partial t^{2}}=-f(x,t)$$

where P(x,t) is the acoustic pressure at a focusing point in the medium at a certain time, *x* and *t* represent the spacial location and the time, respectively, and *f* is the force. By the Fourier transformation and solution, the following formula is received:

(2) $$\int_{S}\left(\bar{P_{2}}\frac{\partial \bar{P_{1}}}{\partial n}-\bar{P_{1}}\frac{\partial \bar{P_{2}}}{\partial n}\right)dS=\int_{V}\left(\bar{P_{1}}\bar{f_{2}}-\bar{P_{2}}\bar{f_{1}}\right)dV$$

Formula (2) is the reciprocal theorem of the wave equation, where *S* is the surface area of the transducer. The relation between any two points’ physical states in the current medium can thus be established, and the transducer’s excited acoustic field can be calculated using the reciprocal theorem in the application of ultrasonic testing.

Firstly, suppose two solutions for the wave Equation (1):

(3) $$\left\{ \begin{array}{l} {\bar{P}}_{1}(x,\omega )=\bar{P}(x,\omega )\\ {\bar{f}}_{1}(x,\omega )=0 \end{array}\right.\left\{ \begin{array}{l} {\bar{P}}_{2}(x,\omega )=\bar{G}(x;y,\omega )\\ {\bar{f}}_{2}(x,\omega )=\delta (x-y) \end{array}\right.$$

where δ(x−y) is the impact response function for point *y*, $\bar{G}(x;y,\omega )$ is the acoustic solution when the impact function acts as the force, also known as the Green’s function. Combined with Formula (2), the result is shown as follows:

(4) $$\int_{V}\bar{P}(x,\omega )\delta (x-y)dy=\int_{S}\left[\bar{G}(x;y,\omega )\frac{\partial \bar{P}(x,\omega )}{\partial n}-\bar{P}(x,\omega )\frac{\partial \bar{G}(x;y,\omega )}{\partial n}\right]dS$$

It can be further simplified into Formula (5) by the replacement and simplification of *x* and *y*.

(5) $$\bar{P}(x,\omega )=\int_{S}\left[\bar{G}(y;x,\omega )\frac{\partial \bar{P}(y,\omega )}{\partial n}-\bar{P}(y,\omega )\frac{\partial \bar{G}(y;x,\omega )}{\partial n}\right]dS$$

As shown in this formula, the acoustic pressure information of any location in the specimen can be gained by taking in the excited acoustic pressure on the transducer’s surface. In order to calculate the distribution of ultrasonic field in the 3D space, solutions to the Green’s functions $\bar{G}(x;y,\omega )$ and $\frac{\partial \bar{P}(y,\omega )}{\partial n}$ need to be worked out:

(6) $$\bar{G}(x;y,\omega )=\frac{\exp(-jkr)}{4\pi r}$$

where *r* represents the distance between point $x=(x_{1},x_{2},x_{3})$ and fixed point $y=(y_{1},y_{2},y_{3})$ in the 3D space. By the simplification of Newton’s Second Law and the Fourier transformation, Formula (7) is shown below:

(7) $$\frac{\partial \bar{P}}{\partial n}=-j\omega \rho_{0}\bar{V_{n}}$$

Suppose V=∇φ and φ is the velocity potential, Formula (8) is obtained:

(8) $$\nabla P=-\rho_{0}\frac{\nabla \varphi }{\partial t}\Rightarrow \bar{P}=-j\omega \rho_{0}\bar{\varphi }$$

Take solution Formulas (6) to (8) to Formula (5) and conduct the inverse Fourier transformation, the result is as follows:

(9) $$\varphi (x,t)=\sqrt{\frac{c}{t}}⊗\int_{S_{T}}\frac{V_{n}(t-\frac{r}{c})}{4\pi r}dS_{T}(t)$$

where $V_{n}$ is the stimulus of the array element and $S_{T}$ is the array element’s area. The radiation acoustic field of a certain array element can be obtained by taking $V_{n}$ and $S_{T}$ to Formula (9). For the calculation of the focus acoustic field of the transducer containing *N* array elements at any point in the radiated space, Formula (10) can be used to superpose the acoustic pressures of every array elements to obtain the total acoustic pressure of the synthetic acoustic beam.

(10) $$p(x,t)=\sum_{i=1}^{N}\varphi (x,t)$$

**Figure 1:**
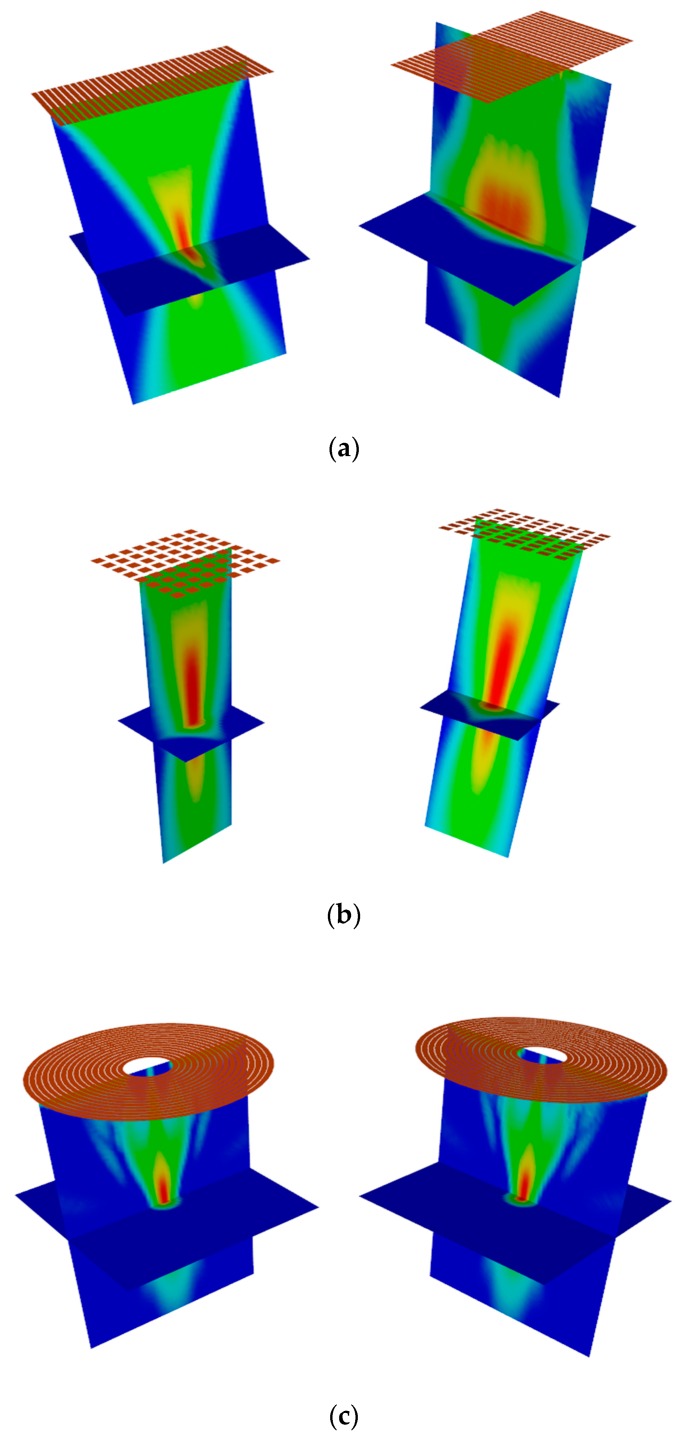
The focus acoustic field of different ultrasonic array transducers in the monolayer medium: (**a**) linear array, (**b**) matrix array, (**c**) annular array.

Figure 1 shows the 3D acoustic field distributions in the 50 mm focused deep monolayer medium of the 5 MHZ 32-element linear array, 64-element matrix array, and 16-element annular array, respectively. It has been found out that the ultrasonic linear array transducer has stronger beam focusing energy along the direction of the elements arrangement, but the energy distribution of the synthetic beam in space is asymmetrical due to the failure of energy focusing along the elements length. Although the matrix ultrasonic transducer can achieve focusing in three dimensions, the focal length is longer and the main lobe energy is relatively insufficient. In addition, the linear and matrix ultrasonic array transducers normally adopt more elements, leading to greater data volume, which is unfavorable for automatic scanning and real-time scanning imaging. The results have shown that the annular array transducer can realize the optimized excited acoustic field using fewer elements, thus, improving the test resolution of materials with high attenuation and large thickness.

### 2.2. TFM Imaging Algorithm of Annular Array

The TFM is a post-processing imaging algorithm based on the full matrix data acquisition and it can achieve arbitrary multiple focusing points in an area [16]. Generally, the TFM imaging area of a linear array transducer is a 2D section, and it is difficult to realize real-time TFM C-scan detection based on the linear ultrasonic transducer under most of the existing hardware conditions because of the large amount of full matrix data. In this paper, based on the axial focusing characteristic of the annular array transducer in the 3D space, we set the single detection area as the linear area along the axis of the transducer, and conduct the pointwise visual focusing in order to achieve the infinite focus of this area.

**Figure 2:**
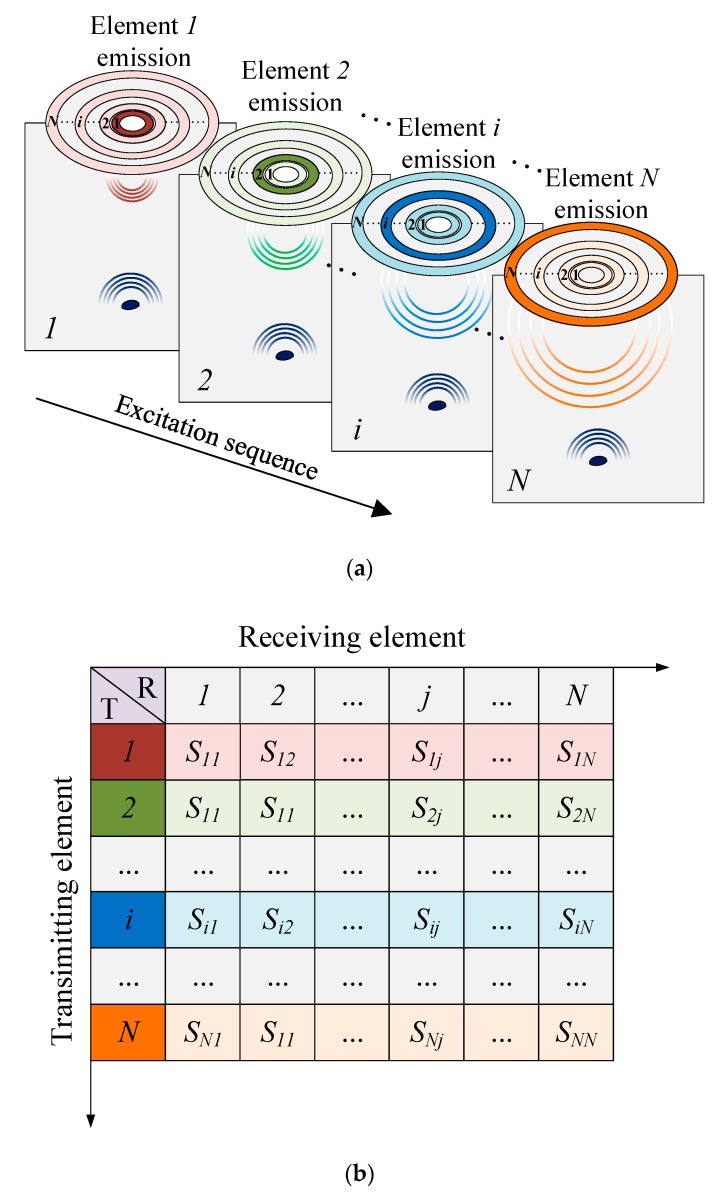
Full matrix data capturing of annular array transducer; (**a**) schematic of data capturing, (**b**) full matrix data obtained from N elements.

For the single detection area, the annular array transducer excites each element in turn, and all elements receive echo signals as well as keeping the signal receipt data. We take turns treating all elements in the annular array transducer as emit–receive units, and gather ultrasonic echo time domain signals, including transmitting the element sequence, receiving the element sequence and the 3D data of time sampling points, i.e., full matrix data [17]. For the ultrasonic board with parallel independent receiving channels, the full matrix data gathering process is similar to that of the linear array transducer. The process is shown as follows: firstly, Element *1* in the annular transducer is excited and all element chips start to receive echo signals as shown in Figure 2a. Then, we define the gathered time-domain ultrasonic echo signal as $S_{1r}(N)$, where *r* = 1, 2, ⋯, *N*. The signal contains the amplitudes of every time sampling point’s received signal. There are *N* groups of data, that is, a row of data in Figure 2b. Finally, based on the above steps, each element in the annular array transducer is excited and N*N groups of echo data are obtained. Since there are fewer elements in the annular array transducer, the time used and the quantity of data is far less than that of other transducers.

**Figure 3:**
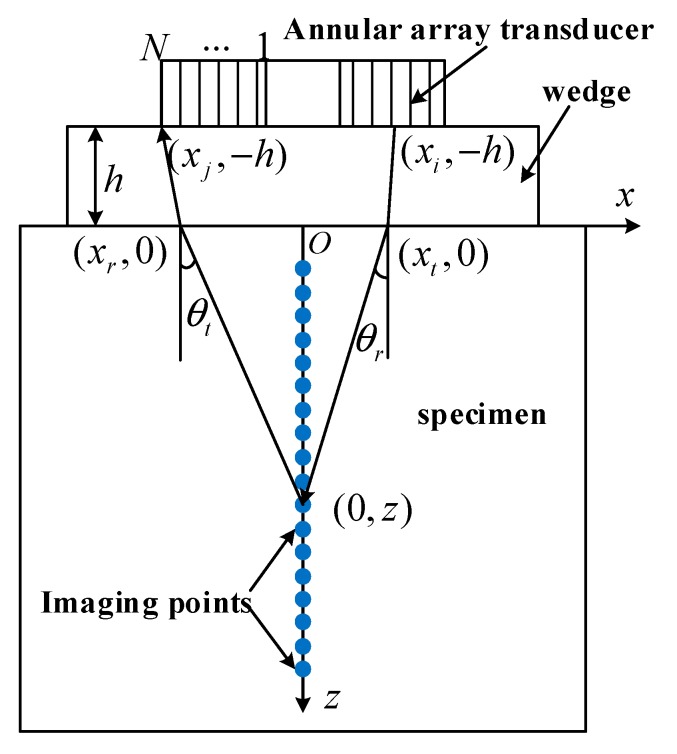
The testing method of total focusing imaging for the annular array transducer.

The full matrix data gathered can be used to visually focus any point on the central axis and achieve the image representation using the synthesized amplitude information. For the regular rectangle specimen and wedge, the algorithm of annular array TFM is rationalized in Figure 3. The surface center of the test specimen is chosen as O, the 2D rectangular coordinate Oxz is set up. Through the gathered full matrix data and the time of propagation, the amplitude of every discrete point on the axis is superposed; thus, the imaging information of every depth along the specimen’s internal axis is gained.

For any point (0,z) on the axis, we ensure that the ultrasonic wave is emitted to point ($x_{t},0$) on the specimen from Element i and returns to the point ($x_{r},0$) on the Element j by the Fermat’s principle, and h is the height of the coupling medium. The total time needed for the propagation is

(11) $$t(0,z)=\frac{\sqrt{{\left(x_{i}-x_{t}\right)}^{2}+h^{2}}+\sqrt{{\left(x_{j}-x_{r}\right)}^{2}+h^{2}}}{c_{1}}+\frac{\sqrt{x_{t}{}^{2}+z^{2}}+\sqrt{x_{r}{}^{2}+z^{2}}}{c_{2}}$$

where $c_{1}$ is the acoustic velocity of the coupling medium, $c_{2}$ is the acoustic velocity of the specimen, and *h* is the height of coupling wedge. For materials with internal anisotropy, the acoustic velocity inside the specimen varies with the propagation angle. Relative $c_{2}$ values from different points can be taken in to optimize the TFM algorithm. 

In order to improve the imaging accuracy, when the hardware permits, the central axis is discretized into as many focusing points as possible to achieve the infinite focusing in the depth direction. For a target focusing point, namely, the detection point, all emit–receive units’ ultrasonic echo signals in the annular array transducer are superposed at this point. The amplitude I(0,z) of the focusing point (0,z) is thus obtained:

(12) $$I\left(0,z\right)=\sum_{i=1}^{N}\sum_{j=1}^{N}S_{ij}(t_{ij}(0,z))$$

where $S_{ij}(t_{ij}(x,z))$ is the amplitude of the representation target point excited by Element i and received by Element j. Therefore, we can obtain the amplitude of every visual focusing point in the detection area through the scanning and complete the information of the whole detection area in the specimen.

### 2.3. Group Velocity Measurement and Anisotrope Analysis

**Figure 4:**
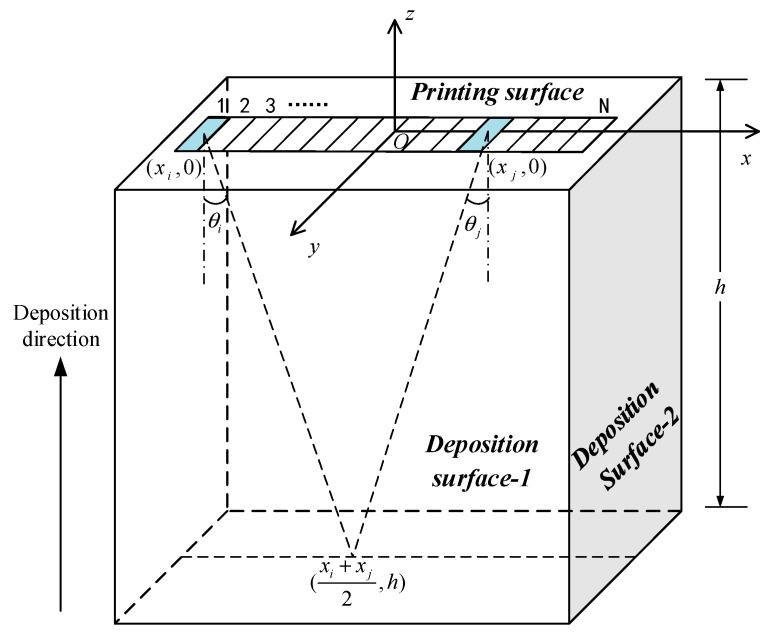
The principle of group velocity measurement based on the full matrix capture method.

The particular features of AM cause heterogeneity in the addictive material’s interior [18]. The overall anisotrope in the AM titanium alloy is caused by the cooling time variation of each printing layer and the growing direction of crystal grains, which leads to the deflection and distortion of the acoustic wave and thus affects the imaging quality. The variation trend of ultrasonic group velocity with different propagating angles in the AM titanium alloy specimen was measured using the full matrix capture method based on the 64-element linear array transducer. [19]. We define the element center as the coordinate system origin *O*. The measurement schematic diagram is shown in Figure 4 by taking the example of group velocity variation in direction *x* on the printing surface. Direction *z* in the figure refers to the deposition direction of the specimen’s manufacturing.

When the Element $x_{i}$ emits the acoustic wave and Element $x_{j}$ receives, the reflection point 1 on the bottom is $\left\{1/2(x_{i}+x_{j}),h\right\}$, where *h* is its thickness in the direction *z*. The propagation angle of the acoustic wave at this moment is

(13) $$\theta_{i}=\theta_{j}=\tan^{-1}\frac{\left|x_{i}-x_{j}\right|}{2d}$$

As $\left(x_{i}-x_{j}\right)$ can be gained from the transducer’s parameters, with the above thickness *h* and the bottom echo wave’s receiving time $t_{ij}$, we can obtain the acoustic group velocity when the angle is $\theta_{i}$:

(14) $$c_{\theta 1}=\sqrt{\frac{{\left(x_{i}-x_{j}\right)}^{2}+4h^{2}}{t_{ij}}}$$

**Figure 5:**
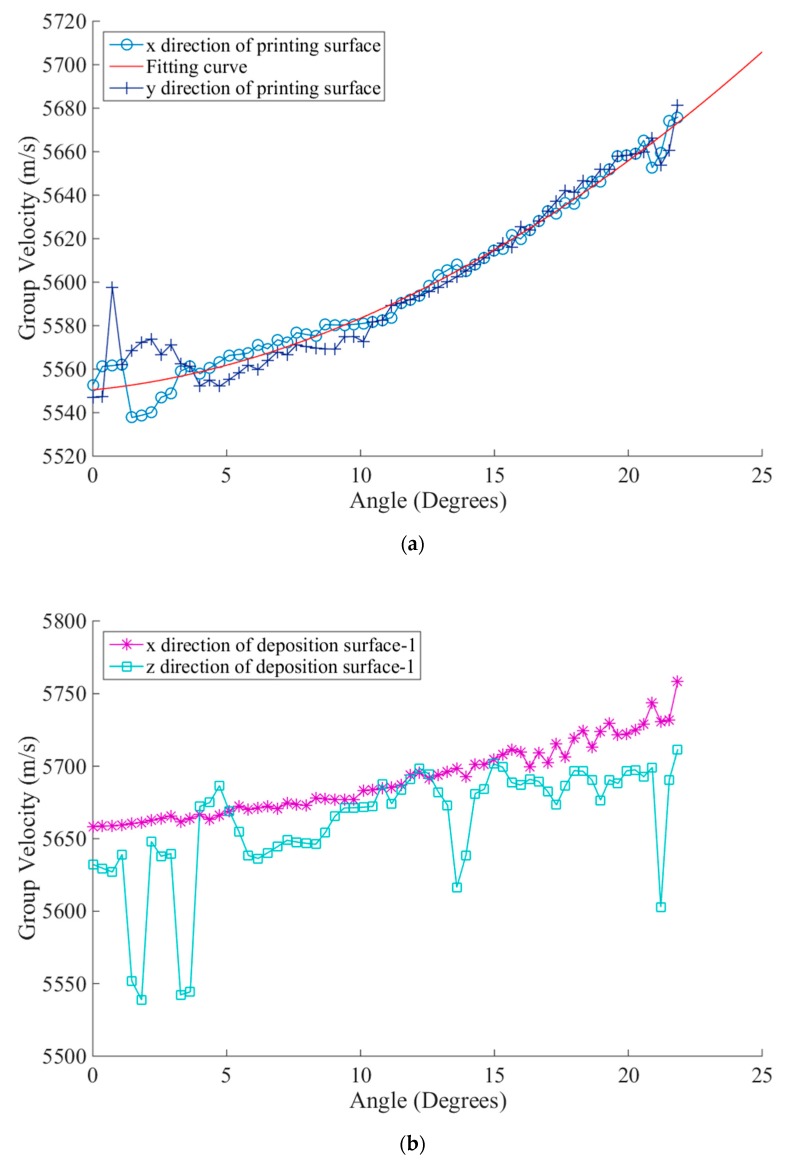

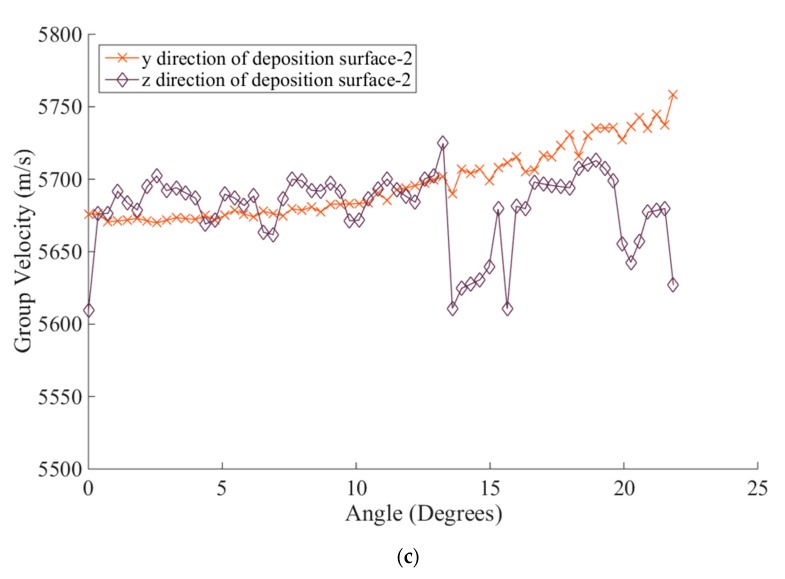
The results of group velocity measurement of different surfaces. (**a**) The variation relationship between the fitting curve of group velocity and angles when the acoustic wave is emitted along the deposition direction. (**b**) and (**c**) are the variation relationship between group velocity and angles when the acoustic wave is emitted perpendicular to the deposition direction.

For the investigation of velocity variation patterns in different directions of the TC 18 AM titanium alloy, we define the specimen’s surface vertical to the deposition direction as the printing surface (*xOy* plane), and the other two adjacent surfaces parallel to the deposition direction as the deposition surface-1 (*xOz* plane) and deposition surface-2 (*yOz* plane). The group velocities of the orthogonal direction on every surface are respectively measured, i.e., direction *x* and *y* on the printing surface, direction *x* and *z* on deposition surface-1, and direction *y* and *z* on deposition surface-2. By the averaging of three measurements in every direction, the results of group velocity is illustrated in Figure 5.

The velocity measurement results in Figure 5a show that when the ultrasonic waves inci along the deposition direction from the printing surface, the group velocity’s variations are relatively regular in two orthogonal directions; all group velocities increase with the increase in angles, and we can use the polyfit method to calculate the group velocity’s variations in the range of different angles and thus optimize the total focusing imaging method. As shown in Figure 5b,c, when the ultrasonic wave is perpendicular to the deposition direction from deposition surface-1 and deposition surface-2, based on the arrangement of elements, the group velocities show relatively more differing variation patterns: when the element is arranged vertical to the deposition direction (*x* direction of deposition surface-1 and *y* direction of deposition surface-2), there are some patterns in the group velocity’s variations. The velocity slightly increases with the increasing angle. When the element arrangement direction corresponds to the deposition direction (*z* direction), there is no obvious pattern in the variations. It is difficult to conduct the fitting. In addition, the group velocity is 100 mm/s when the wave is emitted vertically from the printing surface, higher than that emitted vertically from deposition surface-1 and deposition surface-2. The velocity anisotropy in AM structures is more complex than that in CFRP and other conventional manufactured metal.

## 3. Specimen and Experimental Setup

### 3.1. Specimen

**Figure 6:**
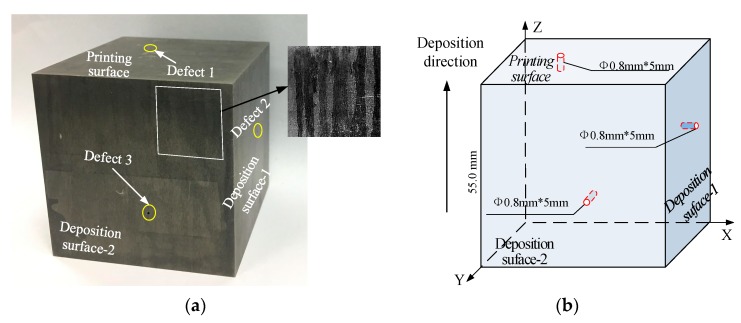
3D-print specimen of TC 18 titanium alloy. (**a**) The actual specimen and defect distribution; (**b**) demonstration of the specimen and defects’ sizes.

The tested object in the confirmatory experiment is a TC18 titanium alloy printed by laser 3D printing technology. The special manufacturing technique makes it distort and attenuate when the ultrasonic travels inside [6]. It is almost impossible to detection with conventional ultrasonic methods. The specimen is a cube with side lengths of 55 mm. On the three adjacent surfaces of the specimen (the printing surface, deposition surface-1, and deposition surface-2), there are flat bottom holes of 0.5 mm deep and 0.8 mm diameter respectively. The holes processed along the deposition direction are marked as Defect 1, and the other two holes are marked as Defect 2 and Defect 3, as shown in Figure 6. When testing from any surface, the vertical projections all include a flat bottom hole defect and two transverse hole defects in different depth and location.

### 3.2. Experimental Setup 

Based on the above inspection theory, we respectively use a linear array transducer and an annular array transducer with the same frequency, and our self-developed ultrasonic array automatic inspection and imaging system to examine the AM TC18 titanium alloy specimen. The parameters for the ultrasonic array transducer are shown in Table 1. The exciting/receiving hardware for testing the ultrasonic wave is the ultrasonic phrased array board produced by the U.S. AOS company. The imaging software is developed by the provided API function. We improve the imaging software’s data processing ability by basing on the GPU with accelerating CUDA paralleling computing; thus, avoiding the point losing in TFM real-time scanning.

**Figure 7:**
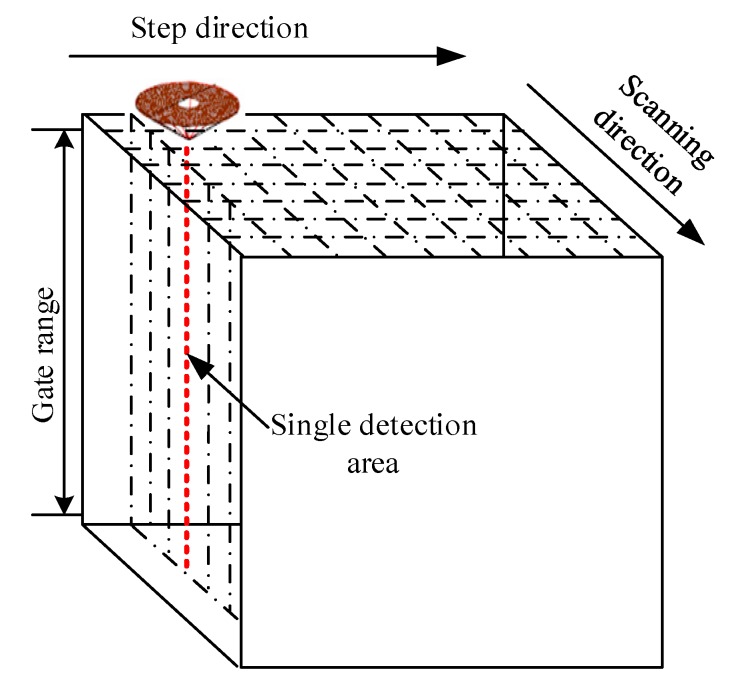
Schematic of the total focusing method (TFM) C-scan inspection using the annular array transducer.

When the linear array transducer is used for detection, we set the aperture size of electronic scanning as 48 to guarantee there is enough beam power, namely, the stepping interval is (64 − 48 + 1) × 0.3 = 5.1 mm, and the stepping resolution is 0.3 mm, equaling the linear array transducer’s element pitch. Additionally, a total of 10 focus points is set at 5 mm intervals from 5 mm to 50 mm above the specimen upper surface when using the conventional DDF method. For the proposed TFM of annular array, we set the discrete spacing of the axis virtual focus points to 0.2 mm. Figure 7 shows the schematic of the specimen’s overall TFM. C-scan imaging is achieved at last by the developed automatic scanning and imaging system. Besides, the relatively large attenuation difference among every location on the specimen surface makes it difficult to detect with the bottom echo [20]. Therefore, the gate range is set as nearly 55 mm to obtain the internal defects echo in the whole specimen, and the amplitude normalization method is used for imaging.

## 4. Results and Discussion

**Figure 8:**
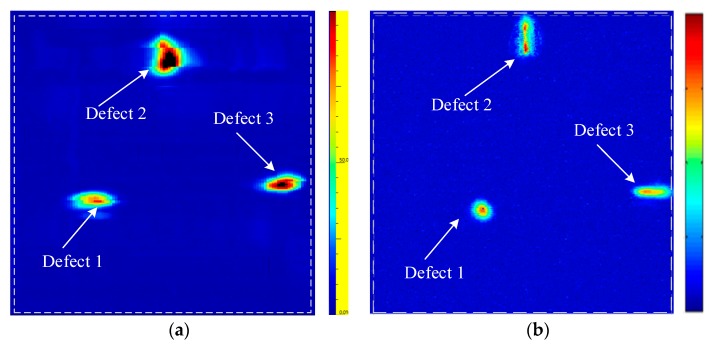
A comparison of linear array and annular array inspection results (the printing surface). (**a**) The linear array C-scan image using the conventional dynamic depth focusing (DDF) method. (**b**) The annular array C-scan image using the TFM.

Figure 8 shows the C-scan result using the printing surface as the beam’s incidence plane. As shown in Figure 8a, there are three obvious defect imaging from the C-scan image when using the electronic scanning method for the linear array transducer. However, due to the asymmetry of ultrasonic energy distribution and the unevenness of focusing ultrasonic energy at different depths, the C-scan results are greatly affected by the acoustic propagation anisotropy and attenuation of the AM titanium alloy material. As a result, the defect in the flat bottom hole appears to be oval and the two transverse holes have different extents of “distortion”. The defects’ imaging sizes are far larger than their actual sizes and it is impossible to represent their shapes accurately. At the same time, the center amplitudes of defects at different depths differ greatly, so it is difficult to evaluate these defects with a uniform method. The TFM C-scan result of the annular array transducer is shown in Figure 8b, where the imaging of a flat bottom hole and two transverse holes is obvious and the positioning of defects is accurate with no distortion. The three defects are measured with −6dB method and all the imaging size errors are under 8%. 

**Figure 9:**
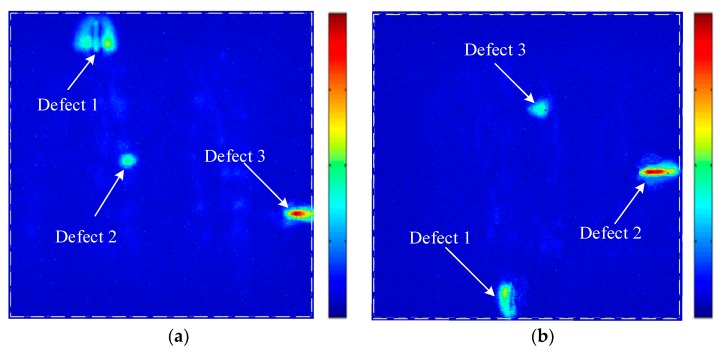
The TFM inspection results of the annular array when the beam is vertical to the deposition direction. (**a**) The C-scan image of deposition surface-1. (**b**) The C-scan image of deposition surface-2.

With the aim to explore how acoustic characteristics of the AM specimen influences the scanning result, experiments on deposition surface-1 and deposition surface-2 as the incidences surface are conducted using the same annular array transducer parameters. Results are shown in Figure 9. All three prefabricated defects can be identified. However, defect-1 in Figure 9a,b all distort in the widths. There are two reasons: the first is when the acoustic wave is incident from the deposition surface-1 and deposition surface-2, as shown in the measuring results in the Figure 5b,c, the group velocity’s variations with the angle demonstrate relatively larger difference in the scanning and stepping direction. It is difficult to optimize by a particular group velocity corrector formula. The second reason is the thick columnar crystal in the AM titanium alloy growing along the deposition direction [21], as shown in Figure 6a. When the wave is emitted vertical to the crystal grain’s growing direction, the more that the grain’s interfaces aggravate the beam’s scattering and distortion. The beam’s attenuation is more serious than that when the wave travels along the crystal grain’s direction. Therefore, compared to the Figure 8b incident from the printing surface, the imaging SNR in the Figure 9 is slightly worse.

**Figure 10:**
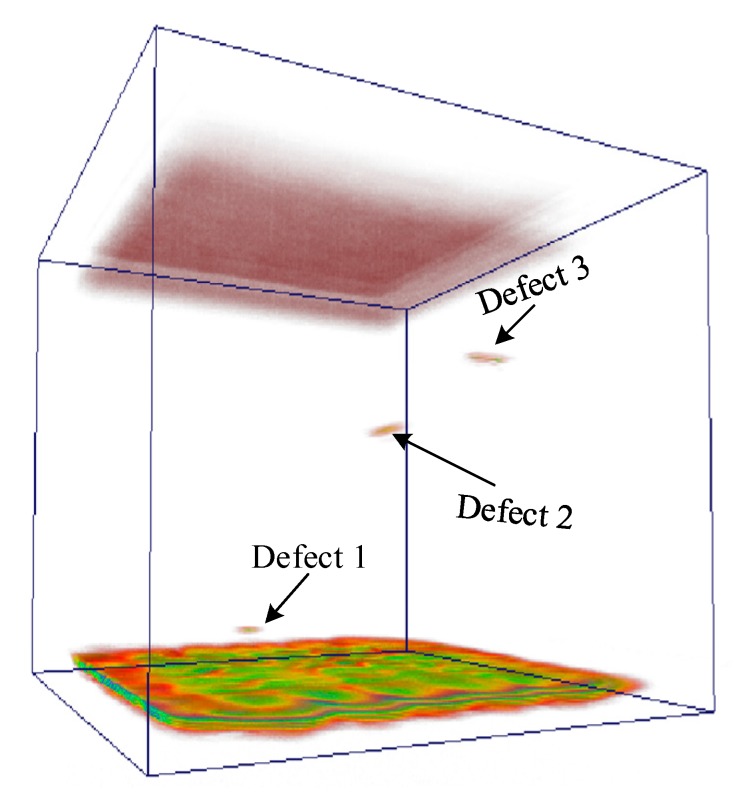
3D imaging result based on the annular array TFM data.

As shown in the above results, the TFM imaging method of annular array transducer can obtain better inspection results of TC18 AM titanium alloy. It can represent the features of defects at different depths accurately and directly and the TFM data can help with the 3D imaging of the whole specimen, as shown in Figure 10. Meanwhile, the element size of the annular transducer is larger and there are fewer elements, so the calculated amount of TFM real-time scanning is far less than that of the linear array transducer. The stronger beam focusing energy and the higher detection accuracy makes it feasible for the inspection of high attenuation material with an uneven internal, such as the AM titanium alloy components.

## 5. Conclusions

(1). The annular ultrasonic array transducer can produce stronger focusing beam energy with fewer elements and the elements are completely symmetrical in space. The proposed TFM imaging algorithm takes advantage of the acoustic field of the annular array transducer effectively, and the data calculation amount is small, thus, it is more suitable for real-time C-can imaging. However, there is still a need to further increase its scanning speed to meet industrial detection applications.

(2). When the acoustic wave is incident from different surfaces of the TC18 titanium alloy, the group velocity’s variations with the angle differ greatly. When the wave is incident along the AM deposition direction, the group velocity’s variations with the angle are relatively regular, and the imaging algorithm can be calibrated with a specific fitting curve formula. If the imaging algorithm cannot be optimized by measuring the wave velocity, it may affect the accuracy of defect imaging of AM titanium alloy components.

(3). Due to the anisotropy and high attenuation characteristics of the AM titanium alloy material, the energy distribution at different depths and the symmetry of the transducer’s focused acoustic field have a great influence on the C-scan results when using internal defect echo to detect. The TFM C-scan results of the annular array transducer can detect the prefabricated defect information at the specimen’s different depths and locations more accurately. The indication is that the proposed TFM of annular array transducer works better for AM titanium alloy and materials with large thickness and high attenuation.

(4). The anisotropy in the AM material is closely related to the growing direction of its internal crystal grains. From the TC18 AM titanium alloy’s C-scan results, we can see that it is necessary to consider the AM material’s deposition direction when using the ultrasonic method. It is suggested to emit the beam along the deposition direction when using the ultrasonic array method for detection.

## Figures and Tables

**Table 1:** Parameters of the array transducer.

Type	Frequency	Element Number	Element Pitch	Element Width
Linear array transducer	10 MHz	64	0.3 mm	0.2 mm
Annular array transducer	10 MHz	16	1.3 mm	1.2 mm

## References

[1] Arcella F.G., Froes F.H. (2000). Producing titanium aerospace components from powder using laser forming. JOM.

[2] Günther D., Heymel B., Günther J.F., Ederer I. (2014). Continuous 3D-printing for additive manufacturing. Rapid Prototyp. J..

[3] SAE International (2011). Titanium Alloy Direct Deposited Products Ti-6Al-4V Annealed.

[4] Hooreweder B.V., Moens D., Boonen R., Kruth J.P., Sas P. (2012). Analysis of fracture toughness and crack propagation of Ti6Al4V produced by selective laser melting. Adv. Eng. Mater..

[5] Kobryn P.A., Moore E.H., Semiatin S.L. (2000). Effect of laser power and traverse speed on microstructure, porosity, and build height in laser-deposited Ti-6Al-4V. Scr. Mater..

[6] Bermingham M., McDonald S.D., Dargusch M.S., St John D.H. (2007). Microstucture of cast titanium alloys. Mater. Forum.

[7] Zeltmann S.E., Gupta N., Tsoutsos N.G., Maniatakos M., Rajendran J., Karri R. (2016). Manufacturing and Security Challenges in 3D Printing. JOM.

[8] Rieder H., Spies M., Bamberg J., Henkel B. (2015). On and Offline Ultrasonic Characterization of Components Built by SLM Additive Manufacturing.

[9] Escobar-Ruiz E., Alejandro E. (2014). Linear and Non-Linear Ultrasonic NDE of Titanium Diffusion Bonds.

[10] Cerniglia D., Scafidi M., Pantano A., Rudlin J. (2015). Inspection of additive-manufactured layered components. Ultrasonics.

[11] Waller J.M., Parker B.H., Hodges K.L., Burke E.R., Walker J.L. (2014). Nondestructive Evaluation of Additive Manufacturing State of the Discipline Report.

[12] Drinkwater B.W., Wilcox P.D. (2006). Ultrasonic arrays for non-destructive evaluation: A review. NDT E Int..

[13] Guan X., Zhang J., Rasselkorde E.M., Abbasi W.A., Zhou S.K. (2014). Material damage diagnosis and characterization for turbine rotors using three-dimensional adaptive ultrasonic NDE data reconstruction techniques. Ultrasonics.

[14] Song S.J., Shin H.J., Jang Y.H. (2002). Development of an ultrasonic phased array system for non-destructive tests of nuclear power plant components. Nucl. Eng. Des..

[15] Schmerr L.W. (2015). Fundamentals of Ultrasonic Phased Arrays.

[16] Yan D., Sutcliffe M., Wright B., Cooper I. (2013). Ultrasonic imaging of full matrix capture acquired data for carbon fibre-reinforced polymer. Insight Non Destr. Test. Cond. Monit..

[17] Zhang J., Drinkwater B.W., Wilcox P.D., Hunter A.J. (2010). Defect detection using ultrasonic arrays: The multi-mode total focusing method. NDT E Int..

[18] Turó A., Chávez J.A., García-Hernández M.J., Bulkai A., Tomek P., Tóth G., Gironés A., Salazar J. (2013). Ultrasonic inspection system for powder metallurgy parts. Measurement.

[19] Li C., Pain D., Wilcox P.D., Drinkwater B.W. (2013). Imaging composite material using ultrasonic arrays. NDT E Int..

[20] Shi Y.W., Yang P.H., Liang J., Wang Z. Relations among ultrasonic testing results and defect characteristics and material properties of laser additive manufacturing titanium alloy. Proceedings of the 19th World Conference of Non-Destructive Testing.

[21] Lin X. (2003). Columnar to equiaxed transition during alloy solidification. Sci. China Ser. E Technol. Sci..

